# Skin, Hair, and Nail Supplements: Marketing and Labeling Concerns

**DOI:** 10.7759/cureus.12062

**Published:** 2020-12-13

**Authors:** Ariadna C Perez-Sanchez, Evelyne K Tantry, Emily K Burns, Veronica M Perez, Sahana Prabhu, Rajani Katta

**Affiliations:** 1 Internal Medicine, University of Texas Health Science Center at San Antonio, San Antonio, USA; 2 Dermatology, Rice University, Houston, USA; 3 Dermatology, Baylor College of Medicine, Houston, USA; 4 Public Health, Texas A&M University, College Station, USA; 5 Internal Medicine, Baylor College of Medicine, Houston, USA; 6 Dermatology, University of Texas Health Science Center at Houston, Houston, USA

**Keywords:** dietary supplement, hair, hair loss, male pattern hair loss, us fda, supplement, skin health, nail diseases, nutrition and dermatology

## Abstract

Background and objective

Dietary supplements advertised to “boost collagen” or for “skin, hair, and nail” health are becoming increasingly popular, despite a lack of evidence to support their use. These products are not regulated by the United States (U.S.) Food and Drug Administration (FDA), and hence there is no centralized database listing current products. The goal of this study was to document and examine the labeling and marketing methods of these products.

Methods

Supplements including the words “glow,” “beauty,” “skin,” “hair,” or “nails” on the label were included in the sample. Seven stores within a 3-mile radius were included.

Results

A total of 176 unique supplements were identified. It was found that most products lacked independent testing; many utilized outdated daily values (DVs) of nutrients. Some had confusing dosing instructions, and most products made health-related marketing claims.

Conclusion

Dermatologists and primary care providers should be aware of the marketing claims commonly made by these products. Patients should be educated that these claims are generally not verified by independent testing agencies, the U.S. FDA, or by high-quality randomized control trials.

## Introduction

Dietary supplements are widely used in the United States (U.S.) and worldwide, with studies estimating that over 50% of the U.S. population regularly consumes some form of a dietary supplement [1]. Within this category, supplements for purported skin, hair, and nail benefits, also known as "dermatology" or "beauty" supplements, are becoming increasingly popular, with the global beauty supplement market expected to reach $7 billion by 2024 [2].

Despite their widespread use, there is a lack of high-quality evidence to support the use of many dermatology supplements, due to a lack of large-scale randomized control trials [3]. No U.S. Food and Drug Administration (FDA) approval is required to produce and market a dietary supplement. For manufacturers, there is no need to show proof of safety or efficacy prior to sale [4]. Since no approval is required, there is no centralized database of currently available dietary supplements. In a previous study published in 2020, we documented the safety concerns related to dietary supplements advertised as skin, hair, nail, and beauty supplements available for sale in local retailers [5]. In this analysis, our goal is to evaluate the labeling and marketing claims of these products and identify any potential areas of confusion for consumers.

## Materials and methods

Details of the study design and methods have been described in a previous report [5]. Briefly, dermatology supplements were defined as those featuring the words “skin,” “hair,” “nails,” “beauty,” or “glow” in the product name or tagline. Seven stores including grocery, drug, department, and cosmetics stores were surveyed within a 3-mile radius of a Houston dermatology practice from August to December 2019. Data were extracted from the label and packaging of each product.

## Results

A total of 176 supplements were identified. Overall, these utilized 255 distinct active ingredients.

Daily values

The U.S. FDA requires that Supplement Facts display the daily values (DVs) for nutrients. These values were first established in 1968. New DVs were established in 2016 and are required to be listed on all product labels by 2021 [6]. It was found that as many as 44 products (25%) in this sample displayed old DVs, rather than the DVs established in 2016.

Dosing instructions

In our sample, 12 products (7%) used a range of dosing. For example, dosage instructions could read, “take 1 tablespoon 1-2 times daily.” Some labels provided serving sizes that did not correspond to the DVs on the Supplement Facts label. One product contained two separate Supplement Facts labels.

Quality testing seals

Products were examined for the presence of seals indicating third-party quality testing by organizations recognized by the U.S. Office of Dietary Supplements, including the U.S. Pharmacopeial Convention (USP) and the National Sanitation Foundation (NSF). Six products (3.4%) displayed such seals.

Allergen information

While there is no U.S. FDA requirement regarding allergen disclosure, many products included some allergen information. Among the sample, 69% indicated that they were “gluten-free” and 48% indicated “no artificial flavors.” One product included the term “gluten-free” on the label and also listed “wheat” in the ingredients, with a notice stating, “wheat has been processed to allow this to the meet the FDA requirements for gluten-free products.”

Among collagen products, 10 labels listed “fish” as the source. Of these 10 products containing fish, nine lacked allergen warnings.

Health marketing claims

The products in our sample made a wide variety of health-related claims. Table 1 provides an overview of the health marketing claims made by supplements. The most common claim was “may help promote healthy hair, skin, and nails” (32% of products) followed by “beauty from within” or “beauty from the inside out” (21%). Other common claims were “maintain or promote skin hydration” (16%), “promote younger looking skin” (16%), “hair, skin, and nail support” (12%), “supports synthesis of collagen in the skin” (11%), and “combat fine lines and wrinkles” (10%).

**Table 1:** Health marketing claims

Health claim	Number of supplements	Percentage
May help promote healthy hair skin and nails	57	32%
Beauty from the inside out or beauty from within	37	21%
Maintain or support skin hydration	29	16%
Promotes younger looking skin	29	16%
Hair, skin, and nail support	21	12%
Supports synthesis of collagen in the skin	19	11%
Combat fine lines and wrinkles	18	10%
Boost your body’s collagen production	15	9%
Improve skin appearance	14	8%
None	14	8%
Promotes strong, thick, and beautiful hair	12	7%
Clinically proven	10	6%
Restore and maintain youthful levels of collagen	11	6%
Skin revitalization	11	6%
Skin elasticity	10	6%
Promotes hair growth	6	4%
Nourishes thinning hair	3	2%
Strengthen nails	4	2%
Drinkable beauty	4	2%

Other marketing claims

Other marketing claims are listed in Table 2. The most common claim was “gluten-free” (69% of products). Other common claims were “no artificial flavors” (48%), “dairy-free” (40%), “preservative-free” (30%), and “no synthetic dyes” (28%).

**Table 2:** Other claims GMO: genetically modified organism

Claim	Number of supplements	Percentage
Gluten-free	121	69%
No artificial flavors	85	48%
Dairy-free	71	40%
Preservative-free	53	30%
No synthetic dyes	50	28%
Non-GMO	48	27%
Vegan/vegetarian	37	21%
Sugar-free	36	20%
Made in the USA	29	16%
No yeast	28	16%

Disclaimer statements

Per the U.S. FDA, if a structure/function claim is made by a supplement, then the manufacturer is required to provide the following disclaimer: “This statement has not been evaluated by the FDA. This product is not intended to diagnose, treat, cure, or prevent any disease.” In our sample, 11 products did not display this disclaimer. Seven of the products lacking a disclaimer made claims that would be considered either health-related (no disclaimer required) or structure/function (disclaimer required). These claims are listed in Table 3.

**Table 3:** Claims made by supplements lacking a U.S. FDA disclaimer FDA: Food and Drug Administration; DHT: dihydrotestosterone

Claim
Nourishment for: healthier hair and skin, strong nails
Supports healthy hair, helps block DHT
For stronger, healthier hair
Gorgeous skin, hair, and nails
Hair, nail, skin, and joint support
Age-less skin formula

## Discussion

Among the products included in our survey, many used outdated nutrient reference values. Some product labels provided dosing instructions that made it challenging to calculate nutrient intake. The vast majority of products also lacked evidence of quality testing. 

Claims on many of the products were also of concern. Because manufacturers are allowed to make any health and/or structure/function claims they choose, contingent upon a disclaimer statement for structure/function claims, such marketing claims may easily lead to consumer confusion. Also of concern was some products making claims that could be considered structure/function claims without displaying an FDA-mandated disclaimer statement. 

As discussed in a previous analysis of safety concerns regarding dermatology supplements, many products also lacked important warning labels about potential risks related to teratogenicity, drug interactions, nutrient overdosing, and other risks [5].

Outdated reference values

In our sample, 25% of products displayed DVs established 50 years ago, as opposed to current DVs that reflect recent nutritional science. The U.S. FDA requires that supplement labels display DVs for nutrients. As the population’s nutritional needs and our understanding of nutrition have evolved over the past 50 years, the FDA updated many of the DVs in 2016 [6]. Specifically, 10 nutrients were adjusted to have higher DVs, while 18 were adjusted to have lower DVs. When the new DVs were established in 2016, the U.S. FDA allowed companies until 2020 or 2021 to comply, depending on the annual sales of the manufacturer [6]. Manufacturers with less than $10 million in annual sales have until January 1, 2021, to comply.

Patients should be counseled that although the use of old DVs may technically adhere to current regulations, it is important to recognize that values established over 50 years ago do not reflect current standards.

In addition, some products used outdated units of measurement. One supplement, for example, listed vitamin A in International Units (IU) as opposed to the updated requirement of retinol activity equivalents (RAE).

Confusing dosing instructions

A number of dosing instructions were unclear or would require calculations to determine the true daily intake of nutrients found in the supplement. Twelve products (7%) used a range of dosing, such as “take 1 tablespoon 1-2 times daily.”

Of concern, some products provided serving sizes that did not correspond to DVs. To determine the accurate daily intake, the consumer would have to make calculations. For example, one product informed users that “1 sachet is 2 tablets. Take 2 sachets daily.” However, the serving size on the label was for two tablets. In this case, if a nutrient was listed as providing 100% of the DV, the actual daily intake by a consumer following the package directions would result in the consumption of 200% of the DV.

One bottle contained two distinct Supplement Facts labels, with contradictory reference values. Further investigation by the authors found that the values reported appeared to conform to U.S. and European Union regulations respectively, although this information was not apparent from the label.

Lack of independent testing quality

Supplement manufacturers are expected to adhere to, but do not have to prove, good manufacturing practices [4]. As result, dietary supplements may have inconsistent or even concerning standards for quality.

Inadequate disintegration of tablets, dosing inconsistencies, and doses that do not reflect that which is listed on the label have all been reported [7]. In addition, contamination of products is a significant concern. Dietary supplements have been contaminated with microbes as well as adulterated with prescription medications and heavy metals [7]. Consumer Lab, an independent investigative laboratory, analyzed a total of 15 collagen supplements. They found one product that contained unacceptably high levels of the heavy metal cadmium [8].

In response to these concerns, consumers are advised to seek products that have undergone quality testing by an independent third-party laboratory. Per the Office of Dietary Supplements (ODS) of the National Institutes of Health, products that have undergone such testing may display a “seal of approval.” Three such organizations listed by the ODS are the United States Pharmacopeia (USP), NSF International (formerly the National Sanitation Foundation), and Consumer Lab [9]. These companies confirm the ingredients and doses listed on the label. They also test for contaminants and adulterations.

In our sample, only six supplements (3.4%) displayed such seals. Interestingly, the use of such seals was unrelated to price. One supplement retailed for $3 for a one-month supply and displayed a seal. None of the supplements retailing for over $100 displayed a seal from USP or NSF. 

Health marketing claims: potential for consumer confusion

It is critical that physicians highlight the distinction between evidence-based treatments and health marketing. Many of the supplements we found made marketing claims that could be confusing to the consumer. 

By law, supplements are “not permitted to be marketed for the purpose of treating, diagnosing, preventing, or curing diseases” and therefore may not make disease claims. [10] Per the FDA, by law “manufacturers may make three types of claims for their dietary supplement products: health claims, structure/function claims, and nutrient content claims” [4]. 

In our sample, many products claimed to provide skin, hair, and nail benefits. Yet, the evidence underlying such claims was unclear. The most common vitamin utilized in these products was biotin (57%), and the most common mineral was zinc (28%). A recent review found very few randomized controlled trials supporting the use of these products, and concluded that “current evidence is insufficient to recommend the use of biotin or zinc supplements in dermatology” [3].

Consumers should also be made aware that treatment using dietary supplementation in those with a nutrient deficiency has different effects compared to the effects on those without a nutrient deficiency. In other words, treatment to reverse a deficiency is not the same as supplementation in those with normal baseline levels of a nutrient. Patients with acquired or congenital biotin deficiency, for example, may experience hair loss and would benefit from supplementation. However, no benefit from supplementation has been seen in those with normal baseline biotin levels [11].

A review of the claims in our sample highlights additional areas of potential consumer confusion. One claim in our sample included the word “supports.” Nevertheless, the claim could be interpreted by some consumers as a treatment for disease: “supports clear complexion for people with non-cystic acne.” Therefore, products using this terminology should make it clear that they are not a medical substitute or treatment for acne.

Some products included wording that suggested scientific or clinical evidence to support the claims. Examples included “helps with the appearance of fine lines around the eyes,” “the generation of new, healthy skin tissue,” and “contributes powerful antioxidant activity to help protect the skin internally from oxidative stress and damaging free radicals.” 

Concerns regarding structure/function claims

The FDA requires that if a structure/function claim is made, then a disclaimer is required that the statement has not been evaluated by the FDA [4]. In our sample, we found 11 products that lacked such a disclaimer. Of these, four made no structure/function claims and therefore would not require a disclaimer. The remaining seven supplements, however, made claims that would be considered either health or structure/function claims. If these were indeed structure/function claims, then lack of a disclaimer would be in violation of the U.S. FDA regulations. A structure/function claim is one that describes “the role of a nutrient or dietary ingredient intended to affect the structure or function of the body.” Examples of claims included “hair, nail, skin, and joint support” and “nourishment for healthier hair and skin.” One product made the claim of “supports healthy hair, helps block DHT.”

Limitations of the study

Our study was designed to investigate products sold in local retailers. Therefore, our sample represents only a limited sample of such products for sale. Further research should evaluate products available in other geographic locations as well as supplements sold online.

## Conclusions

Given the limited regulation of the supplement industry, it is imperative that physicians educate patients about the difference between evidence-based treatments and marketing claims. Physicians must be prepared to educate patients on skin, hair, and nail supplements, as the labeling and marketing of such products may lead to confusion. Health marketing claims are common among these products, although these claims are frequently unsubstantiated or based on use in nutrient-deficient states. 

Patients must also be made aware that although legally compliant, dietary supplement labels may not always provide current or evidence-based information. Many supplements in our sample included outdated nutrient reference values from over 50 years ago. In addition, although consumers are typically advised to seek out products that have undergone independent, third-party laboratory analysis, our sample found that very few supplements had undergone such testing. Given the many reported quality concerns with dietary supplements, we advise that consumers seek products that have undergone third-party testing in order to ensure that they contain the doses advertised on the label and are free of adulteration and contamination.
